# Screening and Risk Algorithms for Detecting Pediatric Suicide Risk in the Emergency Department

**DOI:** 10.1001/jamanetworkopen.2025.33505

**Published:** 2025-09-24

**Authors:** Robert H. Aseltine, Shane J. Sacco, Steven Rogers, Fei Wang, Harold Schwartz, Kun Chen

**Affiliations:** 1 Division of Behavioral Sciences and Community Health, UConn Health, Farmington, Connecticut; 2 Center for Population Health, UConn Health, Farmington, Connecticut; 3 Department of Statistics, University of Connecticut, Storrs; 4 Department of Emergency Medicine, Connecticut Children’s Medical Center, Hartford; 5 Department of Pediatrics, School of Medicine, UConn Health, Farmington, Connecticut; 6 Department of Population Health Sciences, Weill Cornell Medicine, New York, New York; 7 Institute of Living, Hartford HealthCare, Hartford, Connecticut

## Abstract

**Importance:**

The Joint Commission requires hospitals and behavioral health care organizations to identify patients at risk of suicide (National Patient Safety Goal 15.01.01). Risk algorithms and in-person screening for suicide risk show utility in identifying patients at risk of suicidal behavior, yet there is little research examining their comparative performance in children and adolescents.

**Objective:**

To assess the performance of suicide risk screening and risk algorithms in identifying the risk of suicide attempts among pediatric patients in the emergency department (ED).

**Design, Setting, and Participants:**

This retrospective cohort study included youths aged 10 to 18 years presenting to the ED of a northeastern US state between September 1, 2019, and August 31, 2021. Patients were screened for suicide risk using the Ask Suicide-Screening Questions survey and the Columbia–Brief Suicide Severity Rating Scale. Electronic health records from this same cohort containing data from May 31, 2017, to the date of their first encounter within this period were extracted to train a risk algorithm. To observe the presence or absence of a suicide attempt, patients were followed up from their first ED encounter for a minimum of 6 months and a maximum of 2.5 years, through March 2, 2022. Data were analyzed from May 2023 to December 2024.

**Exposure:**

Assessments from suicide risk screening and a risk algorithm.

**Main Outcomes and Measures:**

The occurrence of a suicide attempt following a patient’s first suicide risk screening or first visit in the screening period, if not screened.

**Results:**

Among 19 653 patients included in the analysis, the median age was 14.3 (IQR, 12.1-16.2) years, and 10 007 (50.9%) were female. Four hundred ninety-five patients (2.5%) were treated for a suicide attempt. Among patients screening positive for suicide risk in testing samples (mean, 8.1% [95% CI, 7.6%-8.6%]) and patients in the top 8.1% of the distribution on the algorithm, the algorithm correctly identified a mean of 50.7% (95% CI, 47.3%-54.1%) of those who attempted suicide in contrast to 36.5% (95% CI, 31.9%-41.2%) identified by screening. The algorithm uniquely identified 127% more youths who attempted suicide (125) than did screening (55).

**Conclusions and Relevance:**

In this cohort study of pediatric patients, the risk algorithm was superior to screening across all performance metrics and could substantially assist health care organizations’ efforts to meet the Joint Commission’s National Patient Safety Goal to reduce the risk of suicide.

## Key Points

**Question:**

How does the performance of in-person screening compare with risk algorithms in identifying youths at risk of suicide?

**Findings:**

In this cohort study of 19 653 youths, a risk algorithm using patients’ clinical data significantly outperformed universal screening instruments in identifying pediatric patients in the emergency department at risk of subsequent suicide attempts. The risk algorithm uniquely identified 127% more patients with subsequent suicide attempts than screening.

**Meaning:**

These findings suggest that clinical implementation of suicide risk algorithms will improve identification of at-risk patients and may substantially assist health care organizations’ efforts to meet the Joint Commission’s suicide risk reduction requirement.

## Teaser

This cohort study compares the utility of a suicide risk algorithm derived from patients’ historical clinical records with the performance of universal suicide risk screening in identifying risk for future suicide attempts among youths aged 10 to 18 in the emergency department (ED).

## Introduction

Youth suicide is an escalating health problem in the US. Suicide is the second most common cause of death among adolescents in the US and has increased 62% between 2010 and 2020.^1,2,3^ Recent research has shown that health care clinicians are routinely engaged with children at risk of suicide, albeit with limited recognition of their mental health risk. A longitudinal study of 8 Mental Health Research Network health care systems found that nearly 80% of adolescents who died by suicide had received health care during the year prior to death, with nearly 50% having an emergency department (ED) encounter during that time. Only 32% had a visit of any kind with a mental health–related diagnosis code.^4^ These data highlight the opportunity for youth suicide prevention through improved surveillance, detection, and intervention in the health care system.

In recognition of this opportunity, the Joint Commission established National Patient Safety Goal 15.01.01: Reduce the risk for suicide, a required performance element for all accredited hospitals and behavioral health care organizations.^5^ This requires health care organizations to screen patients at risk of suicide using evidence-based tools. Although validated screening tools, such as the Ask Suicide-Screening Questions (ASQ) survey^6^ and Columbia–Brief Suicide Severity Rating Scale (C-BSSRS),^7^ are readily available, they are limited to a point-in-time snapshot, are typically only conducted during clinical encounters, and can generate high rates of false-positive findings, low sensitivity in certain populations, and a lack of concordance across instruments.^8,9,10,11^ A review of extant research performed by the US Preventive Services Task Force concluded that the benefits of suicide risk screening could not be established^12^; however, a 2023 assessment of the ASQ and the Computerized Adaptive Screen for Suicidal Youth have provided much more positive results related to the predictive validity of suicide risk screening.^13^ The Pew Trust reports that most US health care systems have not adopted screening, and among those that have, most only screen patients with identified mental or behavioral health disorders.^14^

As a result of the National Institute of Mental Health’s recent priorities, there are a number of published algorithms using machine learning approaches with clinical data to identify risk of suicidal behavior among adult and pediatric patients.^15,16,17,18,19,20,21,22,23,24,25,26,27^ Such studies have confirmed the importance of prominent clinical risk factors for suicide attempts and death identified in other suicide-related research^28,29,30,31^ and have identified myriad other characteristics associated with suicidal behavior, resulting in greatly improved accuracy compared with previous efforts.^32^ Follow-up of patients completing suicide risk assessments have found that predictive models achieved higher sensitivity and specificity in identifying suicidal behavior than clinical assessments.^33^ Limitations in such methods have been noted, particularly related to the positive predictive values (PPV) in the estimation of suicide mortality.^34^ Despite this limitation, these studies highlight the potential value of risk algorithms in improving risk identification and the possibility of reduced administrative burden and improved efficiency compared with universal screening programs.^35,36^

Three previous studies^36,37,38^ have compared the performance of screening and risk algorithms in detecting risk of suicidal behavior in different patient populations. Although varying substantially in their approaches to screening and risk estimation, all provide evidence of the value of both screening and risk algorithms in detecting patients at risk of suicide. To date, the only study comparing the performance of screening and risk algorithms in a pediatric patient population found screening with the ASQ to perform slightly better than a risk model based on diagnoses derived from a single ED visit.^37^ In the present study, we expanded this nascent line of research by systematically comparing the utility of a suicide risk algorithm derived from patients’ historical clinical records to the performance of universal suicide risk screening in identifying risk for future suicide attempts among pediatric ED patients.

## Methods

This cohort study was approved by the University of Connecticut Health Center and the study site Institutional Review Boards. A Health Insurance Portability and Accountability Act waiver of informed consent was obtained due to the absence of identifying information in the extracted data. We adhered to Strengthening the Reporting of Observational Studies in Epidemiology (STROBE) reporting guideline.

### Study Design and Data Source

This retrospective cohort study included all patients aged 10 to 18 years presenting to the ED of a pediatric medical center in the northeastern US between September 1, 2019, and August 31, 2021. Deidentified electronic health records (EHR) containing data from May 31, 2017, to March 2, 2022, for this patient cohort were extracted to (1) provide as many as 4 years of historical data prior to the screening encounter (or first ED visit within this period if no screening was completed) to train the risk algorithm and (2) to allow for a sufficient follow-up period to observe the presence or absence of a subsequent suicide attempt. EHR data used in the analysis consisted of patient demographic information, suicide risk screening results, and diagnosis codes from the *International Statistical Classification of Diseases, Tenth Revision* (*ICD-10*).

### Measures

Demographic information consisted of patient self-reports of age and sex (male and female), with age recorded at first visit within the recruitment period. Insurance type was obtained from billing records (coded as Medicaid or Medicare vs other payment types). The distribution of self-reported race and ethnicity (coded as Black or African American (hereinafter Black), Hispanic or Latino, White, and other race or ethnicity [including American Indian or Alaska Native, Asian, Native Hawaiian or Other Pacific Islander, other, and unknown]) is presented to provide information on the generalizability of the results. Due to concerns about the potential for bias in risk algorithms of this type,^39^ the results reported herein omitted race and ethnicity from the risk algorithm.

Universal suicide risk screening was conducted in the pediatric ED during the entire study period. Following a 2019 expert consensus clinical pathway,^40^ in-person suicide risk screening included combined results from 2 commonly used self-report measures administered verbally by clinical staff,^41^ the ASQ^6^ and the C-BSSRS.^7^ Patients were first screened for suicide risk using the ASQ, which yielded 3 risk categories: negative, nonacute positive, or acute positive. Patients categorized as having nonacute positive risk on the ASQ were then administered the C-BSSRS to triage these patients into low-risk, moderate-risk, or high-risk categories. Results from the 2 screening assessments were combined to generate 4 categories of risk: minimal (ASQ negative), low (C-BSSRS low risk), moderate (C-BSSRS moderate risk), and high (ASQ acute positive or C-BSSRS high risk).^42^ Patients with missing screening results were coded as low or minimal risk to be consistent with how such patients were treated clinically in this setting. Patients whose screening results placed them in the moderate-risk or high-risk categories were targeted for further clinical assessment and intervention; consequently, we considered these 2 categories to identify at-risk patients in our comparisons with at-risk patients identified by the risk algorithm.

To be consistent with other published suicide risk algorithms,^32^ the primary outcome of interest was the occurrence of a suicide attempt following a patient’s first suicide risk screening or first visit in the screening period, if not screened. Attempts were identified by *ICD-10* intentional self-harm diagnosis codes and code combinations used in previously published work (eTables 1 and 2 in Supplement 1).^23,24,27^ To observe the presence or absence of a suicide attempt, we followed up patients from their first ED encounter for a minimum of 6 months and a maximum of 2.5 years.

### Statistical Analysis

Data were analyzed from May 2023 to December 2024. All analyses were conducted in R, version 4.3.2 (R Program for Statistical Computing),^43^ and our syntax is available at GitHub.^44^ We deployed a previously published modeling procedure (marginal feature screening plus multivariable lasso regression modeling^45^ using the glmnet package^46^) to identify patients at risk of suicide based on variables extracted from clinical records prior to and during screening.^23,24,27^ Variables included age at first screening in the recruitment period (or first ED encounter if not screened), sex, insurance type, the presence or absence of *ICD-10* diagnosis codes (aggregated to 3 digits), and past suicide attempts.

To evaluate the efficacy of these 2 approaches in risk identification, screening results were compared with out-of-sample estimates made by the risk algorithm. We randomly partitioned the data into 10-folds whereby the algorithm was trained using 90% of the data, and both methods were then tested in the remaining 10%. We repeated the experiment 10 times, each with a different fold of data serving as the testing set. For each approach, the area under the receiver operating characteristics curve (AUROC), area under the precision-recall curve (AUPRC), sensitivity, specificity, PPV, and negative predictive value (NPV) were calculated from each testing set, and the mean was calculated. We provide estimated 95% CIs from testing sets assuming an approximate normal distribution. To provide a fair comparison between the risk algorithm and screening, we defined the at-risk group identified by the risk algorithm to be equal to the percentage of patients determined to be at risk by screening in testing sets. Out-of-sample estimates across testing sets were then examined and true and false classifications of suicide attempts by the algorithm and screening were compared.

To characterize the differences between the at-risk patients identified by the algorithm or screening, we compared the most common diagnosis codes among patients labeled by either approach via McNemar tests.^47^ To determine whether the immediacy of risk (eg, the time to an encounter of an attempt) differed among those categorized as at risk by the algorithm alone, the screening result alone, or both, the median time between screening and event visits were compared using pairwise Wilcoxon rank sum tests. Cumulative incidence curves were also compared with pairwise log-rank tests; the proportional hazards assumption was checked using the Grambsch-Therneau test. *P* values for multiple comparisons were adjusted using the Benjamini-Hochberg method.^48^ Two-sided α = .05 indicated statistical significance.

## Results

A total of 19 653 patients visited the ED between September 2019 and August 2021, of whom 495 (2.5%) were treated for a suicide attempt through March 2022. The study population included 10 007 (50.9%) female and 9646 (49.1%) male patients; the median age was 14.3 (IQR, 12.1-16.2) years. In terms of race and ethnicity, 3111 (15.8%) were Black, 6655 (33.9%) were Hispanic or Latino, 7839 (39.9%) were White, and 2048 (10.4%) were of other race or ethnicity. Table 1 contrasts the characteristics of the study population by those subsequently attempting suicide. Compared with those who did not attempt suicide, suicide attempts were more likely among female (351 [70.9%] vs 9656 [50.4%]) (*P* < .001), White (236 [47.7%] vs 7603 [39.7%]) (*P* = .005), and publicly insured (307 [62.0%] vs 10 172 [53.1%]) (*P* < .001) patients. A total of 2321 patients (11.8%) were not screened due to staff deviation from the screening protocol, medical instability, or developmental inappropriateness, yielding 17 332 patients screened (88.2%). Noncompletion rates were substantially lower among those who attempted suicide (12 [2.4%] vs 2309 [12.1%]; *P* < .001). Overall, 1587 patients (8.1%) had a positive screen result for moderate or high risk of suicide, of whom 181 (36.6%) had a subsequent suicide attempt, in contrast to 1406 (7.3%) of those who did not attempt suicide (*P* < .001). The median observation windows prior to and following screening (ie, the duration of the period from patients’ most distant patient encounter to screening, and from screening to the end of data collection) were longer among those with suicide attempts (eg, those who attempted suicide had a median observation window of 423 [IQR, 0-664] days prior to screening vs 59 [IQR, 0-669] days in those who did not attempt suicide; *P* < .001).

**Table 1.** Patient Characteristics

Characteristic	Patient group, No. (%)			*P* value
	Total (N = 19 653)	Individuals who attempted suicide (n = 495)	Individuals who did not attempt suicide (n = 19 158)	
Sex				
Female	10 007 (50.9)	351 (70.9)	9656 (50.4)	<.001
Male	9646 (49.1)	144 (29.1)	9502 (49.6)	
Age, median (IQR), y	14.3 (12.1-16.2)	14.5 (12.9-15.8)	14.2 (12.1-16.2)	.32
Race and ethnicity^a^				
Black	3111 (15.8)	69 (13.9)	3042 (15.9)	.005
Hispanic or Latino	6655 (33.9)	148 (29.9)	6507 (34.0)	
White	7839 (39.9)	236 (47.7)	7603 (39.7)	
Other^b^	2048 (10.4)	42 (8.5)	2006 (10.5)	
Medicaid or Medicare vs commercial				
Medicaid or Medicare	10 479 (53.3)	307 (62.0)	10 172 (53.1)	<.001
Commercial	9174 (46.7)	188 (38.0)	8986 (46.9)	
Screening results^c^				
Not completed	2321 (11.8)	12 (2.4)	2309 (12.1)	<.001
Negative, minimal or low risk	15 745 (80.1)	302 (61.0)	15 443 (80.6)	
Positive, moderate or high risk	1587 (8.1)	181 (36.6)	1406 (7.3)	
Look back or follow-up period, median (IQR), d				
Prescreening	80 (0-669)	423 (0-664)	59 (0-669)	<.001
Postscreening	617 (386-802)	759 (542-858)	613 (382-799)	<.001

^a^ Based on participant self-report and included as information on the generalizability of the results.

^b^ Includes American Indian or Alaska Native, Asian, Native Hawaiian or Other Pacific Islander, other reported race, and unknown.

^c^ Minimal risk indicates Ask Suicide-Screening Questions (ASQ) negative; low risk, Columbia–Brief Suicide Severity Rating Scale (C-BSSRS) low risk; moderate risk, C-BSSRS moderate risk; and high risk, ASQ acute positive or C-BSSRS high risk.

### Comparison of Screening and the Risk Algorithm

Table 2 presents the relative performance of the risk algorithm and screening in correctly identifying patients at risk of a suicide attempt across 10 splits of the data (eTable 3 in Supplement 1 provides model risk factors). When the cutoff point for the at-risk group on the risk algorithm was set to the percentage of patients with positive screen results (mean, 8.1% [95% CI, 7.6%-8.6%]), the algorithm correctly identified a mean of 50.7% (95% CI, 47.3%-54.1%) of those who attempted suicide in contrast to 36.5% (95% CI, 31.9%-41.2%) identified by screening. The algorithm had a mean AUROC of 0.84 (95% CI, 0.83-0.85), AUPRC of 0.19 (95% CI, 0.16-0.22), sensitivity of 0.51 (95% CI, 0.47-0.54), specificity of 0.93 (95% CI, 0.93-0.94), PPV of 0.16 (95% CI, 0.15-0.17), and NPV of 0.99 (95% CI, 0.99-0.99). In contrast, screening had substantially lower performance on 4 of the 6 metrics, with a mean AUROC of 0.72 (95% CI, 0.70-0.74), AUPRC of 0.06 (95% CI, 0.05-0.06), sensitivity of 0.37 (95% CI, 0.32-0.41), and PPV of 0.11 (95% CI, 0.10-0.12). Differences across all performance metrics were statistically significant as indicated by the fact that the 95% CIs for differences in Table 2 do not include zero.

**Table 2.** Screening and Risk Algorithm Performance Over 10 Data Splits Given the Same Number of Patients Labeled at Risk

Measure	Percentage of patients, mean (95% CI)		
	Screening	Algorithm	Difference
Labeled at risk, %	8.1 (7.6-8.6)	8.1 (7.6-8.6)	NA
AUROC	0.72 (0.70-0.74)	0.84 (0.83-0.85)	0.12 (0.10-0.15)
AUPRC	0.06 (0.05-0.06)	0.19 (0.16-0.22)	0.13 (0.10-0.16)
Sensitivity	0.37 (0.32-0.41)	0.51 (0.47-0.54)	0.14 (0.10-0.18)
Specificity	0.93 (0.92-0.93)	0.93 (0.93-0.94)	<0.01 (<0.01 to <0.01)
PPV	0.11 (0.10-0.12)	0.16 (0.15-0.17)	0.05 (0.03-0.06)
NPV	0.98 (0.98-0.98)	0.99 (0.99-0.99)	<0.01 (<0.01 to <0.01)

Abbreviations: AUROC, area under the receiver operator characteristics curve; AUPRC, area under the precision-recall curve; NA, not applicable; NPV, negative predictive value; PPV, positive predictive value.

**Figure 1.**
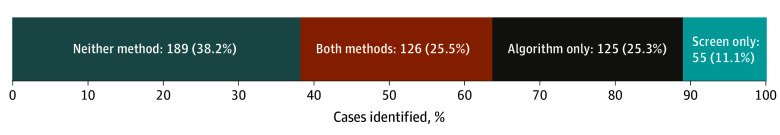
Comparison of Individuals Who Attempted Suicide Identified by Screening and the Risk Algorithm

When combining patients’ risk classifications from screening with those from the risk algorithm, 306 of 495 subsequent individuals who attempted suicide (61.8%) were correctly identified (Figure 1). Classifications from screening and the risk algorithm were in agreement for 126 of these patients (41.2%). Screening uniquely identified 55 of 495 individuals who attempted suicide (11.1%), in contrast to the risk algorithm’s unique identification of 125 (25.3%).

In Table 3, we present the 30 most prevalent historical diagnosis codes among patients categorized as at risk by the risk algorithm and contrast their rank with patients screening positive. There was substantial similarity in the rank ordering of diagnoses codes among those with positive findings on the risk algorithm and on the screening. The top diagnosis codes among patients categorized as at risk by either the algorithm or screening consisted of major depressive disorder, symptoms regarding emotional state, other anxiety disorders, personal risk factors, symptoms regarding appearance and behavior, and prior suicide attempts. Compared with patients screening positive, patients categorized as at risk on the algorithm were more likely to have received a diagnosis across most of the 30 most prevalent diagnosis codes. Further analyses (eTable 4 in Supplement 1) revealed that patients categorized as at risk by the algorithm had a greater number of previous medical encounters and had received more diagnoses across those encounters than those screening positive. Patients deemed at risk by the algorithm had a median of 3 (IQR, 1-7) visits and 11 (IQR, 8-18) diagnoses prior to or at the screening encounter, which was significantly higher than those deemed at risk by the screening (2 [IQR, 1-4] visits and 8 [IQR, 5-12] diagnoses; *P* < .001). These results were similar when only correctly identified individuals who attempted suicide and uniquely identified individuals who attempted suicide by each method were compared.

**Table 3.** Top 30 Historical Diagnosis Codes of Patients Categorized as at Risk by the Algorithm and Their Rank Among Patients With Positive Screen Results

Most prevalent *ICD-10* code	Patients labeled at risk				*P* value
	Algorithm (n = 1587)		Screening (n = 1587)		
	No. (%)	Rank	No. (%)	Rank	
F32: major depressive disorder, single episode	1382 (87.1)	1	1250 (78.8)	1	<.001
R45: symptoms and signs involving emotional state	1314 (82.8)	2	1236 (77.9)	2	.13
F41: other anxiety disorders	944 (59.5)	3	828 (52.2)	3	<.001
Z91: personal risk factors, not elsewhere classified	835 (52.6)	4	586 (36.9)	4	<.001
R46: symptoms and signs involving appearance and behavior	685 (43.2)	5	347 (21.9)	6	<.001
Prior suicide attempt	647 (40.8)	6	432 (27.2)	5	<.001
F90: attention-deficit hyperactivity disorders	586 (36.9)	7	297 (18.7)	9	<.001
Y92: place of occurrence of the external cause	582 (36.7)	8	347 (21.9)	6	<.001
F43: reaction to severe stress, and adjustment disorders	567 (35.7)	9	297 (18.7)	9	<.001
Y93: activity codes	492 (31.0)	10	287 (18.1)	11	<.001
Z79: long-term (current) drug therapy	422 (26.6)	11	316 (19.9)	8	<.001
F91: conduct disorders	409 (25.8)	12	131 (8.3)	21	<.001
F34: persistent mood (affective) disorders	370 (23.3)	13	107 (6.7)	24	<.001
X78: intentional self-harm by sharp object	352 (22.2)	14	206 (13.0)	13	<.001
Z62: problems related to upbringing	283 (17.8)	15	167 (10.5)	18	<.001
Z87: personal history of other diseases and conditions	263 (16.6)	16	168 (10.6)	17	<.001
R51: headache	234 (14.7)	17	153 (9.6)	19	<.001
R11: nausea and vomiting	231 (14.6)	18	197 (12.4)	14	.22
R10: abdominal and pelvic pain	226 (14.2)	19	186 (11.7)	16	.11
J45: asthma	220 (13.9)	20	133 (8.4)	20	<.001
Z20: contact with and (suspected) exposure to communicable diseases	216 (13.6)	21	240 (15.1)	12	.25
F33: major depressive disorder, recurrent	198 (12.5)	22	190 (12.0)	15	>.99
R00: abnormalities of heart beat	174 (11.0)	23	130 (8.2)	22	.008
R44: other symptoms and signs with general sensations and perceptions	170 (10.7)	24	121 (7.6)	23	<.001
F98: other behavioral and emotional disorders with onset usually occur in childhood and adolescence	164 (10.3)	25	40 (2.5)	55	<.001
S50: superficial injury of elbow and forearm	160 (10.1)	26	83 (5.2)	28	<.001
S51: open wound of elbow and forearm	142 (8.9)	27	89 (5.6)	26	<.001
F84: pervasive developmental disorders	132 (8.3)	28	79 (5.0)	29	<.001
F12: cannabis-related disorders	119 (7.5)	29	88 (5.5)	27	.04
Z63: other problem relative to primary support group, including family circumstances	108 (6.8)	30	98 (6.2)	25	.87

Abbreviation: *ICD-10*, *International Statistical Classification of Disease, Tenth Revision*.

**Figure 2.**
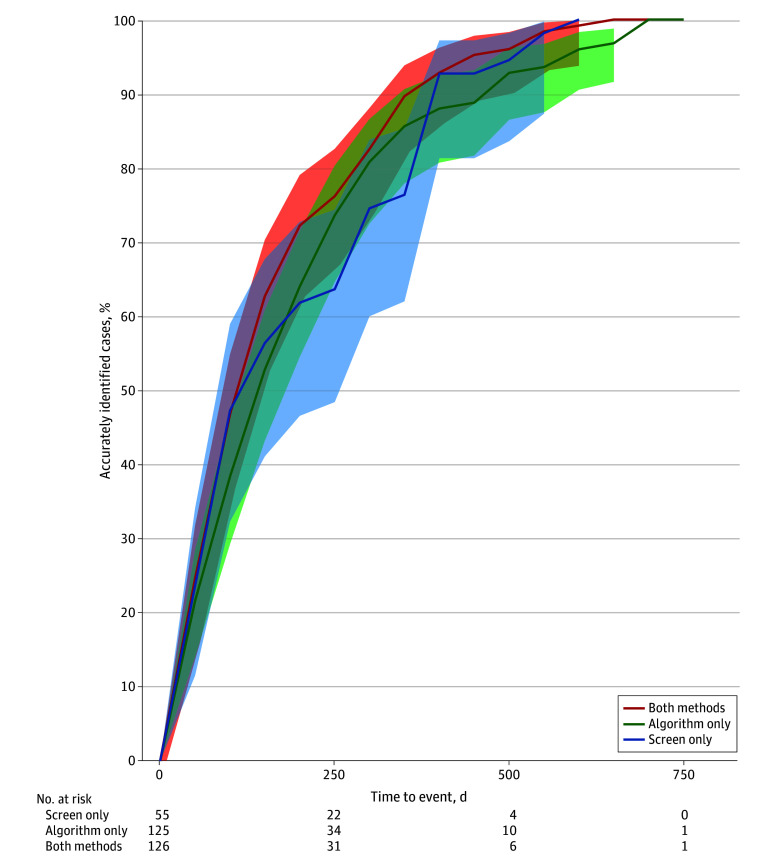
Cumulative Incidence Curves of Accurately Identified Individuals Who Attempted Suicide Shaded areas indicate 95% CIs.

Finally, we estimated the timing of suicide attempts following screening visits among individuals who attempted suicide correctly identified through screening, the risk algorithm, or both methods by calculating the median time to event and cumulative incidence curves (Figure 2). Patients correctly categorized as at risk by both screening and the algorithm had the shortest median time to event of 118 (IQR, 51-242) days or approximately 6 months, but this was not significantly different from individuals who attempted suicide categorized only by screening (109 [IQR, 52-338] days) or only the risk algorithm (143 [IQR, 57-254] days; *P* = .51). Cumulative incidence curves for accurately identified individuals who attempted suicide also did not differ among those categorized by either screening, the algorithm, or both methods (hazard ratios for screen vs algorithm, 1.03 [95% CI, 0.75-1.42]; both methods vs screening, 1.18 [95% CI, 0.86-1.63]; both methods vs algorithm, 1.21 [95% CI, 0.94-1.55]; *P* = .42).

### Post Hoc Analyses

We conducted 2 post hoc sensitivity analyses. To determine whether the risk algorithm capitalized on diagnosis codes generated from the screening encounter, we applied the risk algorithm to patients seen prior to the initiation of universal screening in August 2019. Except for having a lower AUPRC (0.14 [95% CI, 0.12-0.15]; *P* = .002), the mean performance was not significantly different than the results presented in Table 2. Second, we drew on a prior analysis of statewide hospital claims data to determine the likelihood that patients at our study site visited a different hospital during the follow-up period. Most patients (18 264 of 20 197 [90.4%]) had either no visit (15 539 of 20 197 [76.9%]) or returned only to this hospital (2725 of 20 197 [13.5%]) in the follow-up period. eAppendixes 1 and 2 in Supplement 1 provide full details on these analyses.

## Discussion

Our cohort study of 19 653 patients visiting the study ED within a 2-year period found that a suicide risk algorithm was superior to a screening protocol using the ASQ and C-BSSRS in identifying pediatric ED patients at risk of suicide attempts. Using a risk cutoff established by the positive screening result rate in this study population (8.1%), the algorithm labeled 45% more true-positive results (PPV, 0.16 vs 0.11), captured 38% more individuals who attempted suicide (sensitivity, 0.51 vs 0.37), and did not differ from screening in its ability to identify patients at imminent risk of suicidal behavior. The algorithm appeared to benefit from the richness of clinical data available, as those identified by the algorithm had more prior visits and a great number of diagnosis codes than those identified by screening.

One noteworthy aspect of our study was its incorporation of standard practices in both approaches used to identify patients at risk of suicidal behavior. The performance of the risk algorithm was comparable to previously published pediatric suicide risk algorithms,^23,24,27,37^ and the screening protocol drew on measures with established validity and reliability^6,7^ and was implemented with high staff adherence rates. Consequently, these findings cannot be attributed to either unusually good performance of the risk algorithm or deficiencies in patient screening efforts.

One notable criticism of suicide risk models concerns their low PPVs and high rates of false-positive results.^34^ While improvements in model performance are clearly possible, Joint Commission requirements that hospitals and behavioral health organizations proactively identify patients at risk of suicide makes these models clinically appealing despite their current limitations, given their performance relative to current practices. Moreover, our model achieved sensitivities and PPVs at 95% specificity, exceeding the cost-effectiveness thresholds for targeting evidence-based treatments to patients at risk of suicide by Ross et al.^49^

### Limitations

There are limitations to our analysis. First, this study was conducted at a single clinical site, which may limit the generalizability of our findings. Second, we were unable to track patient visits to other ED settings following their screening encounter. However, in examining a prior cohort, we found that more than 90% of patients did not visit another hospital during an identical length of follow-up period, providing some evidence that our findings are unlikely to be biased substantially by differences in utilization patterns. Third, the study cohort received care during the initial wave of the COVID-19 pandemic, a period of increased risk for youth suicide accompanied by changes in the patterning of and risk factors for suicidal behavior.^50^ Fourth, although there is substantial evidence that self-harm events and suicide attempts captured in clinical records and administrative data are associated with subsequent suicide death,^22,51^ determination of suicide attempts using *ICD-10* codes may lead to an undercount of suicide-related events.^52,53^ Previous studies^54,55^ indicate that natural language processing might address limitations in structured EHR data for both risk stratification and risk identification.

## Conclusions

The findings of this cohort study of pediatric ED patients may serve as an impetus for further research to advance best practices in suicide risk identification. Potential permutations in coupling screening and risk algorithms should be investigated, including varying risk cutoffs, screening approaches that incorporate both encounter-based strategies and asynchronous assessment, and the development of risk algorithms that focus on optimizing screening efforts. Such approaches to suicide risk identification hold potential for reducing clinical burden and improving accuracy, allowing the limited behavioral health resources available in hospital settings to be targeted to patients with the highest need.^56,57,58^

## Notes

Supplement 1. **eTable 1.** Suicide Behaviors Identification Algorithm Rules **eTable 2.** Full List of *ICD-10* Codes for Suicide Attempt Identification **eTable 3.** Algorithm Estimators Ordered by Selection Frequency and Coefficient Magnitude **eTable 4.** Comparison of the Number of Visits and Diagnosis Codes Before or During and After Screening of Patients Labeled at Risk by the Risk Algorithm and Screening **eAppendix 1.** Performance of the Risk Algorithm When Applied to a Cohort Before the Screening Protocol **eAppendix 2.** Frequency of Patients Seen at the Study Hospital and Subsequently Seen at Another Hospital

Supplement 2.
Data Sharing Statement
