# Supplementary material for: Screening and Risk Algorithms for Detecting Pediatric Suicide Risk in the Emergency Department
Source: JAMA Netw Open. 2025 Sep 24;8(9):e2533505. doi: 10.1001/jamanetworkopen.2025.33505 (PMC12461438; doi:10.1001/jamanetworkopen.2025.33505)
Supplement: Supplement 1. — eTable 1. Suicide Behaviors Identification Algorithm Rules eTable 2. Full List of ICD-10 Codes for Suicide Attempt Identification eTable 3. Algorithm Estimators Ordered by Selection Frequency and Coefficient Magnitude eTable 4. Comparison of the Number of Visits and Diagnosis Codes Before or During and After Screening of Patients Labeled at Risk by the Risk Algorithm and Screening eAppendix 1. Performance of the Risk Algorithm When Applied to a Cohort Before the Screening Protocol eAppendix 2. Frequency of Patients Seen at the Study Hospital and Subsequently Seen at Another Hospital [file jamanetwopen-e2533505-s001.pdf]

Supplementary Online Content

Aseltine RH, Sacco SJ, Rogers S, Wang F, Schwartz H, Chen K. Screening and risk algorithms for detecting pediatric suicide risk in the emergency department. *JAMA Netw Open.* 2025;8(9):e2533505. doi:10.1001/jamanetworkopen.2025.33505

eTable 1. Suicide Behaviors Identification Algorithm Rules
eTable 2. Full List of *ICD-10* Codes for Suicide Attempt Identification
eTable 3. Algorithm Estimators Ordered by Selection Frequency and Coefficient Magnitude
eTable 4. Comparison of the Number of Visits and Diagnosis Codes Before or During and After Screening of Patients Labeled at Risk by the Risk Algorithm and Screening
eAppendix 1. Performance of the Risk Algorithm When Applied to a Cohort Before the Screening Protocol
eAppendix 2. Frequency of Patients Seen at the Study Hospital and Subsequently Seen at Another Hospital

This supplementary material has been provided by the authors to give readers additional information about their work.

© 2025 Aseltine RH et al. *JAMA Network Open*.

eTable 1. Suicide Behaviors Identification Algorithm Rules

Rule	ICD-10 Code Categories
Intentional self-harm	1. Suicide attempt OR<br>2. Intentional self-harm events OR<br>3. Poisoning and toxic effects, intentional self-harm OR<br>4. Asphyxiation, intentional self-harm
Ideation and Injuries or Events	1a. Suicidal ideation<br><br>AND in the same visit:<br><br>1b. Events of undetermined intent OR<br>1c. Poisoning and toxic effects, undetermined intent OR<br>1d. Asphyxiation, undetermined intent OR<br>1e. Injuries including lacerations, contusions, abrasions, wounds, penetration, puncturing, and superficial foreign bodies and injuries
Suicide-related mental disorders and Suicide-related Injuries or Events	1a. Mental health disorders including depression, dysthymia, mania, bipolar, schizophrenia, behavioral disturbances, anxiety (excluding phobias and separation), stress, delusions, psychotic states, and unspecified mental disorders<br><br>AND in the same visit:<br><br>1b. Events of undetermined intent OR<br>1c. Poisoning and toxic eff, undetermined intent OR<br>1d. Asphyxiation, undetermined intent OR<br>1e. Injuries to wrists, head, or neck including lacerations, contusions, abrasions, wounds, penetration, puncturing, and superficial foreign bodies and injuries

© 2025 Aseltine RH et al. *JAMA Network Open*.

eTable 2. Full List of *ICD-10* Codes for Suicide Attempt Identification

Rule	Category	Codes
Rule 1	Suicide attempt, initial encounter	T1491XA
	Event, intentional self-harm, initial encounter	X710XXA; X711XXA; X712XXA; X713XXA; X718XXA; X719XXA; X72XXXA; X730XXA; X731XXA; X732XXA; X738XXA; X739XXA; X7401XA; X7402XA; X7409XA; X748XXA; X749XXA; X75XXXA; X76XXXA; X770XXA; X771XXA; X772XXA; X773XXA; X778XXA; X779XXA; X780XXA; X781XXA; X782XXA; X788XXA; X789XXA; X79XXXA; X80XXXA; X810XXA; X811XXA; X818XXA; X828XXA; X830XXA; X831XXA; X832XXA; X838XXA
	Poisoning, intentional self-harm, initial encounter	T360X2A; T361X2A; T362X2A; T363X2A; T364X2A; T365X2A; T366X2A; T367X2A; T368X2A; T3692XA; T370X2A; T371X2A; T372X2A; T373X2A; T374X2A; T375X2A; T378X2A; T3792XA; T380X2A; T381X2A; T382X2A; T383X2A; T384X2A; T385X2A; T386X2A; T387X2A; T38802A; T38812A; T38892A; T38902A; T38992A; T39012A; T39092A; T391X2A; T392X2A; T39312A; T39392A; T394X2A; T398X2A; T3992XA; T400X2A; T401X2A; T402X2A; T403X2A; T404X2A; T405X2A; T40602A; T40692A; T407X2A; T408X2A; T40902A; T40992A; T410X2A; T411X2A; T41202A; T41292A; T413X2A; T4142XA; T415X2A; T420X2A; T421X2A; T422X2A; T423X2A; T424X2A; T425X2A; T426X2A; T4272XA; T428X2A; T43012A; T43022A; T431X2A; T43202A; T43212A; T43222A; T43292A; T433X2A; T434X2A; T43502A; T43592A; T43602A; T43612A; T43622A; T43632A; T43692A; T438X2A; T4392XA; T440X2A; T441X2A; T442X2A; T443X2A; T444X2A; T445X2A; T446X2A; T447X2A; T448X2A; T44902A; T44992A; T450X2A; T451X2A; T452X2A; T453X2A; T454X2A; T45512A; T45522A; T45602A; T45612A; T45622A; T45692A; T457X2A; T458X2A; T4592XA; T460X2A; T461X2A; T462X2A; T463X2A; T464X2A; T465X2A; T466X2A; T467X2A; T468X2A; T46902A; T46992A; T470X2A; T471X2A; T472X2A; T473X2A; T474X2A; T475X2A; T476X2A; T477X2A; T478X2A; T4792XA; T480X2A; T481X2A; T48202A; T48292A; T483X2A; T484X2A; T485X2A; T486X2A; T48902A; T48992A; T490X2A; T491X2A; T492X2A; T493X2A; T494X2A; T495X2A; T496X2A; T497X2A; T498X2A; T4992XA; T500X2A; T501X2A; T502X2A; T503X2A; T504X2A; T505X2A; T506X2A; T507X2A; T508X2A; T50A12A; T50A22A; T50A92A; T50B12A; T50B92A; T50Z12A; T50Z92A; T50902A; T50992A

© 2025 Aseltine RH et al. *JAMA Network Open*.

	Toxic effect, intentional self-harm, initial encounter	T510X2A; T511X2A; T512X2A; T513X2A; T518X2A; T5192XA; T520X2A; T521X2A; T522X2A; T523X2A; T524X2A; T528X2A; T5292XA; T530X2A; T531X2A; T532X2A; T533X2A; T534X2A; T535X2A; T536X2A; T537X2A; T5392XA; T540X2A; T541X2A; T542X2A; T543X2A; T5492XA; T550X2A; T551X2A; T560X2A; T561X2A; T562X2A; T563X2A; T564X2A; T565X2A; T566X2A; T567X2A; T56812A; T56892A; T5692XA; T570X2A; T571X2A; T572X2A; T573X2A; T578X2A; T5792XA; T5802XA; T5812XA; T582X2A; T588X2A; T5892XA; T590X2A; T591X2A; T592X2A; T593X2A; T594X2A; T595X2A; T596X2A; T597X2A; T59812A; T59892A; T5992XA
	Asphyxiation, intentional self-harm, initial encounter	T71112A; T71122A; T71132A; T71152A; T71162A; T71192A; T71222A; T71232A
Rule 2A	Suicide ideation, initial encounter	R45851
Rule 2B	Event, undetermined intent, initial encounter	Y210XXA; Y211XXA; Y212XXA; Y213XXA; Y214XXA; Y218XXA; Y219XXA; Y22XXXA; Y230XXA; Y231XXA; Y232XXA; Y233XXA; Y238XXA; Y239XXA; Y240XXA; Y248XXA; Y249XXA; Y25XXXA; Y26XXXA; Y270XXA; Y271XXA; Y272XXA; Y273XXA; Y278XXA; Y279XXA; Y280XXA; Y281XXA; Y282XXA; Y288XXA; Y289XXA; Y29XXXA; Y30XXXA; Y31XXXA; Y32XXXA; Y33XXXA
	Poison, undetermined intent, initial encounter	T360X4A; T361X4A; T362X4A; T363X4A; T364X4A; T365X4A; T366X4A; T367X4A; T368X4A; T3694XA; T370X4A; T371X4A; T372X4A; T373X4A; T374X4A; T375X4A; T378X4A; T3794XA; T380X4A; T381X4A; T382X4A; T383X4A; T384X4A; T385X4A; T386X4A; T387X4A; T38804A; T38814A; T38894A; T38904A; T38994A; T39014A; T39094A; T391X4A; T392X4A; T39314A; T39394A; T394X4A; T398X4A; T3994XA; T400X4A; T401X4A; T402X4A; T403X4A; T404X4A; T405X4A; T40604A; T40694A; T407X4A; T408X4A; T40904A; T40994A; T410X4A; T411X4A; T41204A; T41294A; T413X4A; T4144XA; T415X4A; T420X4A; T421X4A; T422X4A; T423X4A; T424X4A; T425X4A; T426X4A; T4274XA; T428X4A; T43014A; T43024A; T431X4A; T43204A; T43214A; T43224A; T43294A; T433X4A; T434X4A; T43504A; T43594A; T43604A; T43614A; T43624A; T43634A; T43694A; T438X4A; T4394XA; T440X4A; T441X4A; T442X4A; T443X4A; T444X4A; T445X4A; T446X4A; T447X4A; T448X4A; T44904A; T44994A; T450X4A; T451X4A; T452X4A; T453X4A; T454X4A; T45514A; T45524A; T45604A; T45614A; T45624A; T45694A; T457X4A; T458X4A; T4594XA; T460X4A; T461X4A; T462X4A; T463X4A; T464X4A; T465X4A; T466X4A; T467X4A; T468X4A; T46904A; T46994A; T470X4A; T471X4A; T472X4A; T473X4A; T474X4A; T475X4A; T476X4A; T477X4A; T478X4A; T4794XA; T480X4A; T481X4A; T48204A;

© 2025 Aseltine RH et al. *JAMA Network Open*.

		T48294A; T483X4A; T484X4A; T485X4A; T486X4A; T48904A; T48994A; T490X4A; T491X4A; T492X4A; T493X4A; T494X4A; T495X4A; T496X4A; T497X4A; T498X4A; T4994XA; T500X4A; T501X4A; T502X4A; T503X4A; T504X4A; T505X4A; T506X4A; T507X4A; T508X4A; T50A14A; T50A24A; T50A94A; T50B14A; T50B94A; T50Z14A; T50Z94A; T50904A; T50994A
	Toxic effect, undetermined intent, initial encounter	T510X4A; T511X4A; T512X4A; T513X4A; T518X4A; T5194XA; T520X4A; T521X4A; T522X4A; T523X4A; T524X4A; T528X4A; T5294XA; T530X4A; T531X4A; T532X4A; T533X4A; T534X4A; T535X4A; T536X4A; T537X4A; T5394XA; T540X4A; T541X4A; T542X4A; T543X4A; T5494XA; T550X4A; T551X4A; T560X4A; T561X4A; T562X4A; T563X4A; T564X4A; T565X4A; T566X4A; T567X4A; T56814A; T56894A; T5694XA; T570X4A; T571X4A; T572X4A; T573X4A; T578X4A; T5794XA; T5804XA; T5814XA; T582X4A; T588X4A; T5894XA; T590X4A; T591X4A; T592X4A; T593X4A; T594X4A; T595X4A; T596X4A; T597X4A; T59814A; T59894A; T5994XA
	Asphyxiation, undetermined intent, initial encounter	T71114A; T71124A; T71134A; T71144A; T71154A; T71164A; T71194A; T71224A; T71234A
	Contusion, laceration, and hemorrhage, initial encounter	S06370A; S06371A; S06372A; S06373A; S06374A; S06375A; S06376A; S06377A; S06378A; S06379A; S06380A; S06381A; S06382A; S06383A; S06384A; S06385A; S06386A; S06387A; S06388A; S06389A
	Contusion and Laceration, initial encounter	S06310A; S06311A; S06312A; S06313A; S06314A; S06315A; S06316A; S06317A; S06318A; S06319A; S06320A; S06321A; S06322A; S06323A; S06324A; S06325A; S06326A; S06327A; S06328A; S06329A; S06330A; S06331A; S06332A; S06333A; S06334A; S06335A; S06336A; S06337A; S06338A; S06339A

© 2025 Aseltine RH et al. *JAMA Network Open*.

	Laceration, initial encounter	S0101XA; S0102XA; S01111A; S01112A; S01119A; S01121A; S01122A; S01129A; S0121XA; S0122XA; S01311A; S01312A; S01319A; S01321A; S01322A; S01329A; S01411A; S01412A; S01419A; S01421A; S01422A; S01429A; S01511A; S01512A; S01521A; S01522A; S0181XA; S0182XA; S0191XA; S0192XA; S0520XA; S0521XA; S0522XA; S0530XA; S0531XA; S0532XA; S0912XA; S11011A; S11012A; S11021A; S11022A; S11031A; S11032A; S1111XA; S1112XA; S1121XA; S1122XA; S1181XA; S1182XA; S1191XA; S1192XA; S15011A; S15012A; S15019A; S15021A; S15022A; S15029A; S15111A; S15112A; S15119A; S15121A; S15122A; S15129A; S15211A; S15212A; S15219A; S15221A; S15222A; S15229A; S15311A; S15312A; S15319A; S15321A; S15322A; S15329A; S162XXA; S21011A; S21012A; S21019A; S21021A; S21022A; S21029A; S21111A; S21112A; S21119A; S21121A; S21122A; S21129A; S21211A; S21212A; S21219A; S21221A; S21222A; S21229A; S21311A; S21312A; S21319A; S21321A; S21322A; S21329A; S21411A; S21412A; S21419A; S21421A; S21422A; S21429A; S2191XA; S2192XA; S2501XA; S2502XA; S25111A; S25112A; S25119A; S25121A; S25122A; S25129A; S2521XA; S2522XA; S25311A; S25312A; S25319A; S25321A; S25322A; S25329A; S25411A; S25412A; S25419A; S25421A; S25422A; S25429A; S25511A; S25512A; S25519A; S25811A; S25812A; S25819A; S2591XA; S26020A; S26021A; S26022A; S2612XA; S2692XA; S27331A; S27332A; S27339A; S27431A; S27432A; S27439A; S2753XA; S2763XA; S27803A; S27813A; S27893A; S29021A; S29022A; S29029A; S31010A; S31011A; S31020A; S31021A; S31110A; S31111A; S31112A; S31113A; S31114A; S31115A; S31119A; S31120A; S31121A; S31122A; S31123A; S31124A; S31125A; S31129A; S3121XA; S3122XA; S3131XA; S3132XA; S3141XA; S3142XA; S31511A; S31512A; S31521A; S31522A; S31610A; S31611A; S31612A; S31613A; S31614A; S31615A; S31619A; S31620A; S31621A; S31622A; S31623A; S31624A; S31625A; S31629A; S31801A; S31802A; S31811A; S31812A; S31821A; S31822A; S31831A; S31832A; S3501XA; S3502XA; S3511XA; S3512XA; S35211A; S35212A; S35221A; S35222A; S35231A; S35232A; S35291A; S35292A; S35311A; S35321A; S35331A; S35341A; S35411A; S35412A; S35413A; S35414A; S35415A; S35416A; S358X1A; S3591XA; S36030A; S36031A; S36032A; S36039A; S36113A; S36114A; S36115A; S36116A; S36123A; S36230A; S36231A; S36232A; S36239A; S36240A; S36241A; S36242A; S36249A; S36250A; S36251A; S36252A; S36259A; S36260A; S36261A; S36262A; S36269A; S3633XA; S36430A; S36438A; S36439A; S36530A; S36531A; S36532A; S36533A; S36538A; S36539A; S3663XA; S36893A; S3693XA; S37031A; S37032A; S37039A; S37041A; S37042A; S37049A; S37051A; S37052A; S37059A; S37061A; S37062A; S37069A; S3713XA; S3723XA; S3733XA; S37431A; S37432A; S37439A; S37531A; S37532A; S37539A; S3763XA; S37813A; S37823A; S37893A; S3793XA; S39021A; S39022A; S39023A;

		S41011A; S41012A; S41019A; S41021A; S41022A; S41029A; S41111A; S41112A; S41119A; S41121A; S41122A; S41129A; S45011A; S45012A; S45019A; S45111A; S45112A; S45119A; S45211A; S45212A; S45219A; S45311A; S45312A; S45319A; S45811A; S45812A; S45819A; S45911A; S45912A; S45919A; S46021A; S46022A; S46029A; S46121A; S46122A; S46129A; S46221A; S46222A; S46229A; S46321A; S46322A; S46329A; S46821A; S46822A; S46829A; S46921A; S46922A; S46929A; S51011A; S51012A; S51019A; S51021A; S51022A; S51029A; S51811A; S51812A; S51819A; S51821A; S51822A; S51829A; S55011A; S55012A; S55019A; S55111A; S55112A; S55119A; S55211A; S55212A; S55219A; S55811A; S55812A; S55819A; S55911A; S55912A; S55919A; S56021A; S56022A; S56029A; S56121A; S56122A; S56123A; S56124A; S56125A; S56126A; S56127A; S56128A; S56129A; S56221A; S56222A; S56229A; S56321A; S56322A; S56329A; S56421A; S56422A; S56423A; S56424A; S56425A; S56426A; S56427A; S56428A; S56429A; S56521A; S56522A; S56529A; S56821A; S56822A; S56829A; S56921A; S56922A; S56929A; S61011A; S61012A; S61019A; S61021A; S61022A; S61029A; S61111A; S61112A; S61119A; S61121A; S61122A; S61129A; S61210A; S61211A; S61212A; S61213A; S61214A; S61215A; S61216A; S61217A; S61218A; S61219A; S61220A; S61221A; S61222A; S61223A; S61224A; S61225A; S61226A; S61227A; S61228A; S61229A; S61310A; S61311A; S61312A; S61313A; S61314A; S61315A; S61316A; S61317A; S61318A; S61319A; S61320A; S61321A; S61322A; S61323A; S61324A; S61325A; S61326A; S61327A; S61328A; S61329A; S61411A; S61412A; S61419A; S61421A; S61422A; S61429A; S61511A; S61512A; S61519A; S61521A; S61522A; S61529A; S65011A; S65012A; S65019A; S65111A; S65112A; S65119A; S65211A; S65212A; S65219A; S65311A; S65312A; S65319A; S65411A; S65412A; S65419A; S65510A; S65511A; S65512A; S65513A; S65514A; S65515A; S65516A; S65517A; S65518A; S65519A; S65811A; S65812A; S65819A; S65911A; S65912A; S65919A; S66021A; S66022A; S66029A; S66120A; S66121A; S66122A; S66123A; S66124A; S66125A; S66126A; S66127A; S66128A; S66129A; S66221A; S66222A; S66229A; S66320A; S66321A; S66322A; S66323A; S66324A; S66325A; S66326A; S66327A; S66328A; S66329A; S66421A; S66422A; S66429A; S66520A; S66521A; S66522A; S66523A; S66524A; S66525A; S66526A; S66527A; S66528A; S66529A; S66821A; S66822A; S66829A; S66921A; S66922A; S66929A; S71011A; S71012A; S71019A; S71021A; S71022A; S71029A; S71111A; S71112A; S71119A; S71121A; S71122A; S71129A; S75011A; S75012A; S75019A; S75021A; S75022A; S75029A; S75111A; S75112A; S75119A; S75121A; S75122A; S75129A; S75211A; S75212A; S75219A; S75221A; S75222A; S75229A; S75811A; S75812A; S75819A; S75911A; S75912A; S75919A; S76021A; S76022A; S76029A; S76121A; S76122A; S76129A; S76221A; S76222A; S76229A; S76321A;

© 2025 Aseltine RH et al. *JAMA Network Open*.

		S76322A; S76329A; S76821A; S76822A; S76829A; S76921A; S76922A; S76929A; S81011A; S81012A; S81019A; S81021A; S81022A; S81029A; S81811A; S81812A; S81819A; S81821A; S81822A; S81829A; S85011A; S85012A; S85019A; S85111A; S85112A; S85119A; S85141A; S85142A; S85149A; S85171A; S85172A; S85179A; S85211A; S85212A; S85219A; S85311A; S85312A; S85319A; S85411A; S85412A; S85419A; S85511A; S85512A; S85519A; S85811A; S85812A; S85819A; S85911A; S85912A; S85919A; S86021A; S86022A; S86029A; S86121A; S86122A; S86129A; S86221A; S86222A; S86229A; S86321A; S86322A; S86329A; S86821A; S86822A; S86829A; S86921A; S86922A; S86929A; S91011A; S91012A; S91019A; S91021A; S91022A; S91029A; S91111A; S91112A; S91113A; S91114A; S91115A; S91116A; S91119A; S91121A; S91122A; S91123A; S91124A; S91125A; S91126A; S91129A; S91211A; S91212A; S91213A; S91214A; S91215A; S91216A; S91219A; S91221A; S91222A; S91223A; S91224A; S91225A; S91226A; S91229A; S91311A; S91312A; S91319A; S91321A; S91322A; S91329A; S95011A; S95012A; S95019A; S95111A; S95112A; S95119A; S95211A; S95212A; S95219A; S95811A; S95812A; S95819A; S95911A; S95912A; S95919A; S96021A; S96022A; S96029A; S96121A; S96122A; S96129A; S96221A; S96222A; S96229A; S96821A; S96822A; S96829A; S96921A; S96922A; S96929A
	Contusion, initial encounter	S0003XA; S0010XA; S0011XA; S0012XA; S0033XA; S00431A; S00432A; S00439A; S00531A; S00532A; S0083XA; S0093XA; S0510XA; S0511XA; S0512XA; S100XXA; S1083XA; S1093XA; S2000XA; S2001XA; S2002XA; S2020XA; S20211A; S20212A; S20219A; S20221A; S20222A; S20229A; S2601XA; S2611XA; S2691XA; S27321A; S27322A; S27329A; S27421A; S27422A; S27429A; S2752XA; S27802A; S27812A; S27892A; S300XXA; S301XXA; S30201A; S30202A; S3021XA; S3022XA; S3023XA; S303XXA; S36020A; S36021A; S36029A; S36112A; S36122A; S36220A; S36221A; S36222A; S36229A; S3632XA; S36420A; S36428A; S36429A; S36520A; S36521A; S36522A; S36523A; S36528A; S36529A; S3662XA; S36892A; S3692XA; S37011A; S37012A; S37019A; S37021A; S37022A; S37029A; S3712XA; S3722XA; S3732XA; S37421A; S37422A; S37429A; S37521A; S37522A; S37529A; S3762XA; S37812A; S37822A; S37892A; S3792XA; S40011A; S40012A; S40019A; S40021A; S40022A; S40029A; S5000XA; S5001XA; S5002XA; S5010XA; S5011XA; S5012XA; S6000XA; S60011A; S60012A; S60019A; S60021A; S60022A; S60029A; S60031A; S60032A; S60039A; S60041A; S60042A; S60049A; S60051A; S60052A; S60059A; S6010XA; S60111A; S60112A; S60119A; S60121A; S60122A; S60129A; S60131A; S60132A; S60139A; S60141A; S60142A; S60149A; S60151A; S60152A; S60159A; S60211A; S60212A; S60219A; S60221A; S60222A; S60229A; S7000XA; S7001XA; S7002XA; S7010XA; S7011XA; S7012XA; S8000XA; S8001XA; S8002XA; S8010XA; S8011XA; S8012XA;

© 2025 Aseltine RH et al. *JAMA Network Open*.

		S9000XA; S9001XA; S9002XA; S90111A; S90112A; S90119A; S90121A; S90122A; S90129A; S90211A; S90212A; S90219A; S90221A; S90222A; S90229A; S9030XA; S9031XA; S9032XA
	Puncture, initial encounter	S0103XA; S0104XA; S01131A; S01132A; S01139A; S01141A; S01142A; S01149A; S0123XA; S0124XA; S01331A; S01332A; S01339A; S01341A; S01342A; S01349A; S01431A; S01432A; S01439A; S01441A; S01442A; S01449A; S01531A; S01532A; S01541A; S01542A; S0183XA; S0184XA; S0193XA; S0194XA; S11013A; S11014A; S11023A; S11024A; S11033A; S11034A; S1113XA; S1114XA; S1123XA; S1124XA; S1183XA; S1184XA; S1193XA; S1194XA; S21031A; S21032A; S21039A; S21041A; S21042A; S21049A; S21131A; S21132A; S21139A; S21141A; S21142A; S21149A; S21231A; S21232A; S21239A; S21241A; S21242A; S21249A; S21331A; S21332A; S21339A; S21341A; S21342A; S21349A; S21431A; S21432A; S21439A; S21441A; S21442A; S21449A; S2193XA; S2194XA; S31030A; S31031A; S31040A; S31041A; S31130A; S31131A; S31132A; S31133A; S31134A; S31135A; S31139A; S31140A; S31141A; S31142A; S31143A; S31144A; S31145A; S31149A; S3123XA; S3124XA; S3133XA; S3134XA; S3143XA; S3144XA; S31531A; S31532A; S31541A; S31542A; S31630A; S31631A; S31632A; S31633A; S31634A; S31635A; S31639A; S31640A; S31641A; S31642A; S31643A; S31644A; S31645A; S31649A; S31803A; S31804A; S31813A; S31814A; S31823A; S31824A; S31833A; S31834A; S41031A; S41032A; S41039A; S41041A; S41042A; S41049A; S41131A; S41132A; S41139A; S41141A; S41142A; S41149A; S51031A; S51032A; S51039A; S51041A; S51042A; S51049A; S51831A; S51832A; S51839A; S51841A; S51842A; S51849A; S61031A; S61032A; S61039A; S61041A; S61042A; S61049A; S61131A; S61132A; S61139A; S61141A; S61142A; S61149A; S61230A; S61231A; S61232A; S61233A; S61234A; S61235A; S61236A; S61237A; S61238A; S61239A; S61240A; S61241A; S61242A; S61243A; S61244A; S61245A; S61246A; S61247A; S61248A; S61249A; S61330A; S61331A; S61332A; S61333A; S61334A; S61335A; S61336A; S61337A; S61338A; S61339A; S61340A; S61341A; S61342A; S61343A; S61344A; S61345A; S61346A; S61347A; S61348A; S61349A; S61431A; S61432A; S61439A; S61441A; S61442A; S61449A; S61531A; S61532A; S61539A; S61541A; S61542A; S61549A; S71031A; S71032A; S71039A; S71041A; S71042A; S71049A; S71131A; S71132A; S71139A; S71141A; S71142A; S71149A; S81031A; S81032A; S81039A; S81041A; S81042A; S81049A; S81831A; S81832A; S81839A; S81841A; S81842A; S81849A; S91031A; S91032A; S91039A; S91041A; S91042A; S91049A; S91131A; S91132A; S91133A; S91134A; S91135A; S91136A; S91139A; S91141A; S91142A; S91143A; S91144A; S91145A; S91146A; S91149A; S91231A; S91232A; S91233A;

© 2025 Aseltine RH et al. *JAMA Network Open*.

		S91234A; S91235A; S91236A; S91239A; S91241A; S91242A; S91243A; S91244A; S91245A; S91246A; S91249A; S91331A; S91332A; S91339A; S91341A; S91342A; S91349A
	Penetrating injuries, initial encounter	S0540XA; S0541XA; S0542XA; S0550XA; S0551XA; S0552XA; S0560XA; S0561XA; S0562XA
	Superficial injuries, initial encounter	S0000XA; S00201A; S00202A; S00209A; S0030XA; S00401A; S00402A; S00409A; S00501A; S00502A; S0080XA; S0090XA; S1010XA; S1080XA; S1090XA; S20101A; S20102A; S20109A; S20301A; S20302A; S20309A; S20401A; S20402A; S20409A; S2090XA; S3091XA; S3092XA; S3093XA; S3094XA; S3095XA; S3096XA; S3097XA; S3098XA; S40911A; S40912A; S40919A; S40921A; S40922A; S40929A; S50901A; S50902A; S50909A; S50911A; S50912A; S50919A; S60391A; S60392A; S60399A; S60911A; S60912A; S60919A; S60921A; S60922A; S60929A; S60931A; S60932A; S60939A; S60940A; S60941A; S60942A; S60943A; S60944A; S60945A; S60946A; S60947A; S60948A; S60949A; S70911A; S70912A; S70919A; S70921A; S70922A; S70929A; S80911A; S80912A; S80919A; S80921A; S80922A; S80929A; S90911A; S90912A; S90919A; S90921A; S90922A; S90929A; S90931A; S90932A; S90933A; S90934A; S90935A; S90936A
	Superficial foreign bodies, initial encounter	S0005XA; S00251A; S00252A; S00259A; S0035XA; S00451A; S00452A; S00459A; S00551A; S00552A; S0085XA; S0095XA; S1015XA; S1085XA; S1095XA; S20151A; S20152A; S20159A; S20351A; S20352A; S20359A; S20451A; S20452A; S20459A; S2095XA; S30850A; S30851A; S30852A; S30853A; S30854A; S30855A; S30856A; S30857A; S40251A; S40252A; S40259A; S40851A; S40852A; S40859A; S50351A; S50352A; S50359A; S50851A; S50852A; S50859A; S60351A; S60352A; S60359A; S60450A; S60451A; S60452A; S60453A; S60454A; S60455A; S60456A; S60457A; S60458A; S60459A; S60551A; S60552A; S60559A; S60851A; S60852A; S60859A; S70251A; S70252A; S70259A; S70351A; S70352A; S70359A; S80251A; S80252A; S80259A; S80851A; S80852A; S80859A; S90451A; S90452A; S90453A; S90454A; S90455A; S90456A; S90551A; S90552A; S90559A; S90851A; S90852A; S90859A

© 2025 Aseltine RH et al. *JAMA Network Open*.

	Other open wounds, initial encounter	S0100XA; S01101A; S01102A; S01109A; S0120XA; S01301A; S01302A; S01309A; S01401A; S01402A; S01409A; S01501A; S01502A; S0180XA; S0190XA; S11019A; S11029A; S11039A; S1110XA; S1120XA; S1180XA; S1189XA; S1190XA; S21001A; S21002A; S21009A; S21101A; S21102A; S21109A; S21201A; S21202A; S21209A; S21301A; S21302A; S21309A; S21401A; S21402A; S21409A; S2190XA; S31000A; S31001A; S31100A; S31101A; S31102A; S31103A; S31104A; S31105A; S31109A; S3120XA; S3130XA; S3140XA; S31501A; S31502A; S31600A; S31601A; S31602A; S31603A; S31604A; S31605A; S31609A; S31809A; S31819A; S31829A; S31839A; S41001A; S41002A; S41009A; S41101A; S41102A; S41109A; S45301A; S45302A; S45309A; S45391A; S45392A; S45399A; S51001A; S51002A; S51009A; S51801A; S51802A; S51809A; S61001A; S61002A; S61009A; S61101A; S61102A; S61109A; S61200A; S61201A; S61202A; S61203A; S61204A; S61205A; S61206A; S61207A; S61208A; S61209A; S61300A; S61301A; S61302A; S61303A; S61304A; S61305A; S61306A; S61307A; S61308A; S61309A; S61401A; S61402A; S61409A; S61501A; S61502A; S61509A; S65201A; S65202A; S65209A; S65291A; S65292A; S65299A; S71001A; S71002A; S71009A; S71101A; S71102A; S71109A; S81001A; S81002A; S81009A; S81801A; S81802A; S81809A; S91001A; S91002A; S91009A; S91101A; S91102A; S91103A; S91104A; S91105A; S91106A; S91109A; S91201A; S91202A; S91203A; S91204A; S91205A; S91206A; S91209A; S91301A; S91302A; S91309A
Rule 3A	Major depressive disorders	F0631; F0632; F32; F320; F321; F322; F323; F324; F325; F328; F329; F33; F330; F331; F332; F333; F334; F3340; F3341; F3342; F338; F339; F4321; F4323
	Bipolar disorders	F31; F310; F311; F3110; F3111; F3112; F3113; F312; F313; F3130; F3131; F3132; F314; F315; F316; F3160; F3161; F3162; F3163; F3164; F317; F3170; F3171; F3172; F3173; F3174; F3175; F3176; F3177; F3178; F3181; F319
	Manic disorders	F0633; F30; F301; F3010; F3011; F3012; F3013; F302; F303; F304; F308; F309
	Other mood disorders	F063; F0630; F0631; F0632; F0633; F0634; F1014; F1024; F1094; F1114; F1124; F1194; F1314; F1324; F1394; F1414; F1424; F1494; F1514; F1524; F1594; F1614; F1624; F1694; F1814; F1824; F1894; F1914; F1924; F1994; F34; F348; F349; F39
	Dysthymia	F341

© 2025 Aseltine RH et al. *JAMA Network Open*.

	Psychotic disorders	F060; F062; F1015; F10150; F10151; F10159; F1025; F10250; F10251; F10259; F1095; F10950; F10951; F10959; F1115; F11150; F11151; F11159; F1125; F11250; F11251; F11259; F1195; F11950; F11951; F11959; F1215; F12150; F12151; F12159; F1225; F12250; F12251; F12259; F1295; F12950; F12951; F12959; F1315; F13150; F13151; F13159; F1325; F13250; F13251; F13259; F1395; F13950; F13951; F13959; F1415; F14150; F14151; F14159; F1425; F14250; F14251; F14259; F1495; F14950; F14951; F14959; F1515; F15150; F15151; F15159; F1525; F15250; F15251; F15259; F1595; F15950; F15951; F15959; F1615; F16150; F16151; F16159; F1625; F16250; F16251; F16259; F1695; F16950; F16951; F16959; F1815; F18150; F18151; F18159; F1825; F18250; F18251; F18259; F1895; F18950; F18951; F18959; F1915; F19150; F19151; F19159; F1925; F19250; F19251; F19259; F1995; F19950; F19951; F19959; F23; F24; F28; F29; F48; F488; F489; F53
	Adjustment disorders	F43; F432; F4320; F4321; F4322; F4323; F4324; F4325; F4329
	Stress disorders	F430; F431; F4310; F4311; F4312; F438; F439
	Schizophrenic disorders	F20; F200; F201; F202; F203; F205; F208; F2081; F2089; F209; F21; F25; F250; F251; F258; F259; F601
	Other Delusional disorders	F22
	Primary hallucination disorders	R440; R441; R442; R443
	Personality disorders	F07; F070; F078; F0789; F079; F60; F600; F602; F603; F604; F605; F606; F607; F608; F6081; F6089; F609; F68; F688; F69
	Behavioral disturbances	F0151; F0281; F0391
	Unspecified mental disorder	F99
Rule 3B	Event, undetermined intent, initial encounter	Y210XXA; Y211XXA; Y212XXA; Y213XXA; Y214XXA; Y218XXA; Y219XXA; Y22XXXA; Y230XXA; Y231XXA; Y232XXA; Y233XXA; Y238XXA; Y239XXA; Y240XXA; Y248XXA; Y249XXA; Y25XXXA; Y26XXXA; Y270XXA; Y271XXA; Y272XXA; Y273XXA; Y278XXA;

© 2025 Aseltine RH et al. *JAMA Network Open*.

		Y279XXA; Y280XXA; Y281XXA; Y282XXA; Y288XXA; Y289XXA; Y29XXXA; Y30XXXA; Y31XXXA; Y32XXXA; Y33XXXA
	Poison, undetermined intent, initial encounter	T360X4A; T361X4A; T362X4A; T363X4A; T364X4A; T365X4A; T366X4A; T367X4A; T368X4A; T3694XA; T370X4A; T371X4A; T372X4A; T373X4A; T374X4A; T375X4A; T378X4A; T3794XA; T380X4A; T381X4A; T382X4A; T383X4A; T384X4A; T385X4A; T386X4A; T387X4A; T38804A; T38814A; T38894A; T38904A; T38994A; T39014A; T39094A; T391X4A; T392X4A; T39314A; T39394A; T394X4A; T398X4A; T3994XA; T400X4A; T401X4A; T402X4A; T403X4A; T404X4A; T405X4A; T40604A; T40694A; T407X4A; T408X4A; T40904A; T40994A; T410X4A; T411X4A; T41204A; T41294A; T413X4A; T4144XA; T415X4A; T420X4A; T421X4A; T422X4A; T423X4A; T424X4A; T425X4A; T426X4A; T4274XA; T428X4A; T43014A; T43024A; T431X4A; T43204A; T43214A; T43224A; T43294A; T433X4A; T434X4A; T43504A; T43594A; T43604A; T43614A; T43624A; T43634A; T43694A; T438X4A; T4394XA; T440X4A; T441X4A; T442X4A; T443X4A; T444X4A; T445X4A; T446X4A; T447X4A; T448X4A; T44904A; T44994A; T450X4A; T451X4A; T452X4A; T453X4A; T454X4A; T45514A; T45524A; T45604A; T45614A; T45624A; T45694A; T457X4A; T458X4A; T4594XA; T460X4A; T461X4A; T462X4A; T463X4A; T464X4A; T465X4A; T466X4A; T467X4A; T468X4A; T46904A; T46994A; T470X4A; T471X4A; T472X4A; T473X4A; T474X4A; T475X4A; T476X4A; T477X4A; T478X4A; T4794XA; T480X4A; T481X4A; T48204A; T48294A; T483X4A; T484X4A; T485X4A; T486X4A; T48904A; T48994A; T490X4A; T491X4A; T492X4A; T493X4A; T494X4A; T495X4A; T496X4A; T497X4A; T498X4A; T4994XA; T500X4A; T501X4A; T502X4A; T503X4A; T504X4A; T505X4A; T506X4A; T507X4A; T508X4A; T50A14A; T50A24A; T50A94A; T50B14A; T50B94A; T50Z14A; T50Z94A; T50904A; T50994A
	Toxic effect, undetermined intent, initial encounter	T510X4A; T511X4A; T512X4A; T513X4A; T518X4A; T5194XA; T520X4A; T521X4A; T522X4A; T523X4A; T524X4A; T528X4A; T5294XA; T530X4A; T531X4A; T532X4A; T533X4A; T534X4A; T535X4A; T536X4A; T537X4A; T5394XA; T540X4A; T541X4A; T542X4A; T543X4A; T5494XA; T550X4A; T551X4A; T560X4A; T561X4A; T562X4A; T563X4A; T564X4A; T565X4A; T566X4A; T567X4A; T56814A; T56894A; T5694XA; T570X4A; T571X4A; T572X4A; T573X4A; T578X4A; T5794XA; T5804XA; T5814XA; T582X4A; T588X4A; T5894XA; T590X4A; T591X4A; T592X4A; T593X4A; T594X4A; T595X4A; T596X4A; T597X4A; T59814A; T59894A; T5994XA
	Asphyxiation, undetermined intent, initial encounter	T71114A; T71124A; T71134A; T71144A; T71154A; T71164A; T71194A; T71224A; T71234A

© 2025 Aseltine RH et al. *JAMA Network Open*.

	Contusion, laceration, and hemorrhage, initial encounter	S06370A; S06371A; S06372A; S06373A; S06374A; S06375A; S06376A; S06377A; S06378A; S06379A
	Contusion and Laceration, initial encounter	S06310A; S06311A; S06312A; S06313A; S06314A; S06315A; S06316A; S06317A; S06318A; S06319A; S06320A; S06321A; S06322A; S06323A; S06324A; S06325A; S06326A; S06327A; S06328A; S06329A; S06330A; S06331A; S06332A; S06333A; S06334A; S06335A; S06336A; S06337A; S06338A; S06339A
	Laceration, initial encounter	S0101XA; S0102XA; S01111A; S01112A; S01119A; S01121A; S01122A; S01129A; S0121XA; S0122XA; S01311A; S01312A; S01319A; S01321A; S01322A; S01329A; S01411A; S01412A; S01419A; S01421A; S01422A; S01429A; S01511A; S01512A; S01521A; S01522A; S0181XA; S0182XA; S0191XA; S0192XA; S0520XA; S0521XA; S0522XA; S0530XA; S0531XA; S0532XA; S0912XA; S1181XA; S1182XA; S1191XA; S1192XA; S162XXA; S26020A; S26021A; S26022A; S2612XA; S2692XA; S36230A; S36240A; S36250A; S36260A; S46121A; S46122A; S46129A; S51811A; S51812A; S51819A; S51821A; S51822A; S51829A; S55011A; S55012A; S55019A; S55111A; S55112A; S55119A; S55211A; S55212A; S55219A; S55811A; S55812A; S55819A; S55911A; S55912A; S55919A; S56021A; S56022A; S56029A; S56121A; S56122A; S56123A; S56124A; S56125A; S56126A; S56127A; S56128A; S56129A; S56221A; S56222A; S56229A; S56321A; S56322A; S56329A; S56421A; S56422A; S56423A; S56424A; S56425A; S56426A; S56427A; S56428A; S56429A; S56521A; S56522A; S56529A; S56821A; S56822A; S56829A; S56921A; S56922A; S56929A; S61511A; S61512A; S61519A; S61521A; S61522A; S61529A; S65011A; S65012A; S65019A; S65111A; S65112A; S65119A; S65811A; S65812A; S65819A; S65911A; S65912A; S65919A; S66021A; S66022A; S66029A; S66120A; S66121A; S66122A; S66123A; S66124A; S66125A; S66126A; S66127A; S66128A; S66129A; S66221A; S66222A; S66229A; S66320A; S66321A; S66322A; S66323A; S66324A; S66325A; S66326A; S66327A; S66328A; S66329A; S66421A; S66422A; S66429A; S66520A; S66521A; S66522A; S66523A; S66524A; S66525A; S66526A; S66527A; S66528A; S66529A; S66821A; S66822A; S66829A; S66921A; S66922A; S66929A; S75011A; S75012A; S75019A; S75021A; S75022A; S75029A; S75111A; S75112A; S75119A; S75121A; S75122A; S75129A
	Contusion, initial encounter	S0003XA; S0010XA; S0011XA; S0012XA; S0033XA; S00431A; S00432A; S00439A; S00531A; S00532A; S0083XA; S0093XA; S0510XA; S0511XA; S0512XA; S1083XA; S1093XA; S2601XA; S2611XA; S2691XA; S36220A; S5010XA; S5011XA; S5012XA; S60211A; S60212A; S60219A

© 2025 Aseltine RH et al. *JAMA Network Open*.

	Puncture, initial encounter	S0103XA; S0104XA; S01131A; S01132A; S01139A; S01141A; S01142A; S01149A; S0123XA; S0124XA; S01331A; S01332A; S01339A; S01341A; S01342A; S01349A; S01431A; S01432A; S01439A; S01441A; S01442A; S01449A; S01531A; S01532A; S01541A; S01542A; S0183XA; S0184XA; S0193XA; S0194XA; S1183XA; S1184XA; S1193XA; S1194XA; S51831A; S51832A; S51839A; S51841A; S51842A; S51849A; S61531A; S61532A; S61539A; S61541A; S61542A; S61549A
	Penetrating injuries, initial encounter	S0540XA; S0541XA; S0542XA; S0550XA; S0551XA; S0552XA; S0560XA; S0561XA; S0562XA
	Superficial injuries, initial encounter	S0000XA; S00201A; S00202A; S00209A; S0030XA; S00401A; S00402A; S00409A; S00501A; S00502A; S0080XA; S0090XA; S1080XA; S1090XA; S50911A; S50912A; S50919A; S60911A; S60912A; S60919A
	Superficial foreign bodies, initial encounter	S0005XA; S00251A; S00252A; S00259A; S0035XA; S00451A; S00452A; S00459A; S00551A; S00552A; S0085XA; S0095XA; S1085XA; S1095XA; S50851A; S50852A; S50859A; S60851A; S60852A; S60859A
	Other open wounds, initial encounter	S0100XA; S01101A; S01102A; S01109A; S0120XA; S01301A; S01302A; S01309A; S01401A; S01402A; S01409A; S01501A; S01502A; S0180XA; S0190XA; S1180XA; S1189XA; S1190XA; S51801A; S51802A; S51809A; S61501A; S61502A; S61509A

© 2025 Aseltine RH et al. *JAMA Network Open*.

eTable 3. Algorithm Estimators Ordered by Selection Frequency and Coefficient Magnitude

Model estimator	Coefficient, M (SD)†	Selection frequency, No.
F32: Major depressive disorder, single episode	0.87 (0.06)	10
Y09: Assault by unspecified means	0.68 (0.20)	10
Z91: Personal risk factors, not elsewhere classified	0.59 (0.03)	10
X78: Intentional self-harm by sharp object	0.57 (0.10)	10
R45: Symptoms and signs involving emotional state	0.54 (0.06)	10
F34: Persistent mood [affective] disorders	0.48 (0.04)	10
F43: Reaction to severe stress, and adjustment disorders	0.42 (0.03)	10
Sex	-0.42 (0.11)	10
Prior suicide attempt	0.37 (0.11)	10
F90: Attention-deficit hyperactivity disorders	0.32 (0.04)	10
R46: Symptoms and signs involving appearance and behavior	0.28 (0.06)	10
Y04: Assault by bodily force	0.27 (0.14)	10
F91: Conduct disorders	0.27 (0.06)	10
Z62: Problems related to upbringing	0.21 (0.05)	10
Z79: Long term (current) drug therapy	0.18 (0.08)	10
F41: Other anxiety disorders	0.18 (0.05)	10
S51: Open wound of elbow and forearm	0.13 (0.07)	10
R44: Oth symptoms and signs w general sensations and perceptions	0.11 (0.08)	10
F98: Oth behav/emotn disord w onset usly occur in chldhd and adol	0.10 (0.05)	10
F40: Phobic anxiety disorders	0.64 (0.30)	9
F29: Unsp psychosis not due to a substance or known physiol cond	0.54 (0.24)	9
F25: Schizoaffective disorders	0.32 (0.15)	9
Z87: Personal history of other diseases and conditions	0.14 (0.05)	9
Z03: Encntr for medical obs for susp diseases and cond ruled out	0.12 (0.09)	9
X71: Intentional self-harm by drowning and submersion	2.24 (0.35)	8
F99: Mental disorder, not otherwise specified	0.77 (0.29)	8
Y07: Perpetrator of assault, maltreatment and neglect	0.48 (0.21)	8
T44: Drugs primarily affecting the autonomic nervous system	0.28 (0.19)	8
T14: Injury of unspecified body region	0.12 (0.12)	8
F63: Impulse disorders	0.18 (0.13)	7
S60: Superficial injury of wrist, hand and fingers	0.15 (0.10)	7
Z72: Problems related to lifestyle	-0.15 (0.14)	6
F84: Pervasive developmental disorders	0.13 (0.07)	6
X83: Intentional self-harm by other specified means	0.09 (0.07)	6
F94: Disord social w onset specific to childhood and adolescence	0.08 (0.04)	6
Age, years	-0.02 (0.01)	6
J06: Acute upper resp infections of multiple and unsp sites	-0.25 (0.15)	5

© 2025 Aseltine RH et al. *JAMA Network Open*.

S50: Superficial injury of elbow and forearm	-0.09 (0.08)	5
Insurance type	0.07 (0.03)	5
F60: Specific personality disorders	0.10 (0.06)	4
S71: Open wound of hip and thigh	0.07 (0.05)	4
F33: Major depressive disorder, recurrent	0.06 (0.04)	4
Z63: Oth prob rel to prim support group, inc family circumstances	-0.19 (0.10)	3
F39: Unspecified mood [affective] disorder	0.17 (0.18)	3
T47: Agents primarily affecting the gastrointestinal system	1.71 (0.28)	2
R09: Oth symptoms and signs involving the circ and resp sys	-0.09 (0.08)	2
T51: Toxic effect of alcohol	0.12 (NA)	1
F12: Cannabis related disorders	0.07 (NA)	1
T65: Toxic effect of other and unspecified substances	0.04 (NA)	1
F31: Bipolar disorder	0.01 (NA)	1
F64: Gender identity disorders	0.00 (NA)	1
Note. †Mean and standard deviation across repetitions.		

© 2025 Aseltine RH et al. *JAMA Network Open*.

eTable 4. Comparison of the Number of Visits and Diagnosis Codes Before or During and After Screening of Patients Labeled at Risk by the Risk Algorithm and Screening

	**Algorithm, median (IQR)**	**Screening, median (IQR)**	***p***
Labeled			
Visits before or during screening	3 (1-7)	2 (1-4)	<0.001
Diagnoses before or during screening	11 (8-18)	8 (5-12)	<0.001
Cases			
Visits before or during screening	4 (2-8)	2 (1-5)	<0.001
Diagnoses before or during screening	14 (9-21)	9 (6-15)	<0.001

© 2025 Aseltine RH et al. *JAMA Network Open*.

eAppendix 1. Performance of the Risk Algorithm When Applied to a Cohort Before the Screening Protocol

To determine if the risk algorithm capitalized on diagnosis codes generated from the screening encounter, we applied the risk algorithm to patients seen prior to the initiation of universal screening in August 2019. For a fair comparison, we matched the length of the recruitment period (730 days), the lookback period for historical records (1,553 days), and the duration of follow-up observations (915 days). There were 456 attempters and 21,975 non-attempters among 10-18 year olds, from which we randomly sampled 17,649 non-attempters to match the prevalence of suicide attempts in the original cohort (2.5%). We calculated area under the ROC curve (AUROC), area under the precision-recall curve (AUPRC), sensitivity, specificity, positive predictive value and negative predictive value, setting the percentage of at-risk patients to match screening results (8.1%). To compare average performance metrics of this prior cohort model to the main cohort model, we conducted a series of two sample t-tests, which revealed that performance did not statistically differ for all metrics (p>0.06), except for AURPC, which was higher in the main cohort model (0.18 vs. 0.14 in prior cohort; p=0.002).

	**Algorithm, mean (95% CI)**	**Versus main cohort model, *p***
AUROC	0.83 (0.80-0.85)	0.07
AUPRC	0.14 (0.12-0.15)	0.002
Sensitivity	0.47 (0.43-0.51)	0.07
Specificity	0.15 (0.13-0.16)	0.58
PPV	0.99 (0.98-0.99)	0.06
NPV	0.93 (0.92-0.93)	0.06

Note. AUROC=area under the ROC curve. AUPRC=area under the precision-recall curve. PPV=positive predictive value. NPV=negative predictive value.

© 2025 Aseltine RH et al. *JAMA Network Open*.

eAppendix 2. Frequency of Patients Seen at the Study Hospital and Subsequently Seen at Another Hospital

Given this was a single-site study, and it was possible that patients may have visited another hospital during follow-up, we examined 2012-2017 statewide admission data of an earlier cohort to approximate the likelihood that these patients were seen at another hospital. For a fair comparison, we matched the length of the recruitment period (730 days) and the duration of follow-up observations (915 days). A total of 20,197 10-18 year old patients were seen at the study hospital during the recruitment period, in which, 4,658 (23.1%) had any visit following their first visit in the recruitment period to the end of the study and 1,966 (9.6%) were seen at another hospital.

© 2025 Aseltine RH et al. *JAMA Network Open*.
