# Supplementary material for: Screening and Risk Algorithms for Detecting Pediatric Suicide Risk in the Emergency Department
Source: JAMA Netw Open. 2025 Sep 24;8(9):e2533505. doi: 10.1001/jamanetworkopen.2025.33505 (PMC12461438; doi:10.1001/jamanetworkopen.2025.33505)
Supplement: Supplement 2. — Data Sharing Statement [file jamanetwopen-e2533505-s002.pdf]

Data Sharing Statement

Aseltine, Jr. Screening and Risk Algorithms for Detecting Pediatric Suicide Risk in the Emergency Department. *JAMA Netw Open*. Published September 24, 2025.
doi:10.1001/jamanetworkopen.2025.33505

Data
Data available: No

Additional Information
Explanation for why data not available: The data consist of pediatric patients clinical records. We have obtained these data under a data use agreement that prohibit us from re-releasing the data.
